# Open questions on the biological roles of first-row transition metals

**DOI:** 10.1038/s42004-020-00341-w

**Published:** 2020-08-07

**Authors:** Debbie C. Crans, Kateryna Kostenkova

**Affiliations:** 1 Department of Chemistry, 1301 Center Ave., Colorado State University, Fort Collins, CO 80523 USA; 2 Cell and Molecular Biology Program, 1301 Center Ave., Colorado State University, Fort Collins, CO 80523 USA

**Keywords:** Metalloproteins, Bioinorganic chemistry, Metals

## Abstract

First-row transition metals play several roles in biological processes and in medicine, but can be toxic in high concentrations. Here the authors comment on the sensitive biochemistry and speciation chemistry of the first-row transition metals, and outline some of the remaining questions that have yet to be answered.

Five of the ten first-row transition metals are essential to human health, including manganese, iron, cobalt, copper, and zinc^1,2^. Three more first-row transition elements have shown some beneficial biological effects including chromium, vanadium, and nickel. Typically, these metals are consumed in a varied diet or as nutritional additives where, in the human body, they serve both structural and functional roles including the maintenance of cellular functions involved in a wide range of biological activities. However, normal function requires that the levels of the metal ions are maintained within an acceptable range; lower concentrations may result in a nutritional deficiency and higher concentrations may result in toxicity (Fig. 1)^3^. In addition, the physical properties of first-row elements, particularly titanium and nickel, are important for preparation of new materials and alloys, resulting in technological advantages that improve the quality of life. Nine of the ten first-row transition metals have densities larger than 5.0 g/cm^3^ which, by some definitions, classifies them as ‘heavy metals’. Although this definition may be commonly used by some, it is not embraced by chemists primarily because this definition depends on the density of the metal rather than its chemical properties. Furthermore, the negative connotation associated with the term ‘heavy metal’ and the toxicity of metals such as cadmium and mercury stands in opposition to the fact that five of the first-row transition elements are essential to life. A more concise definition of the vague term ‘heavy metal’ can be based on chemical properties and would include the block of metals in Groups 3 to 16 that are in periods 4 and greater^4^. This definition of ‘heavy metals’ does not involve first-row elements but only second and third-row transition metals. However, even this definition is debated^4^. It is however clear that none of the five essential first-row transition metals are toxic ‘heavy metals’.

**Fig. 1:**
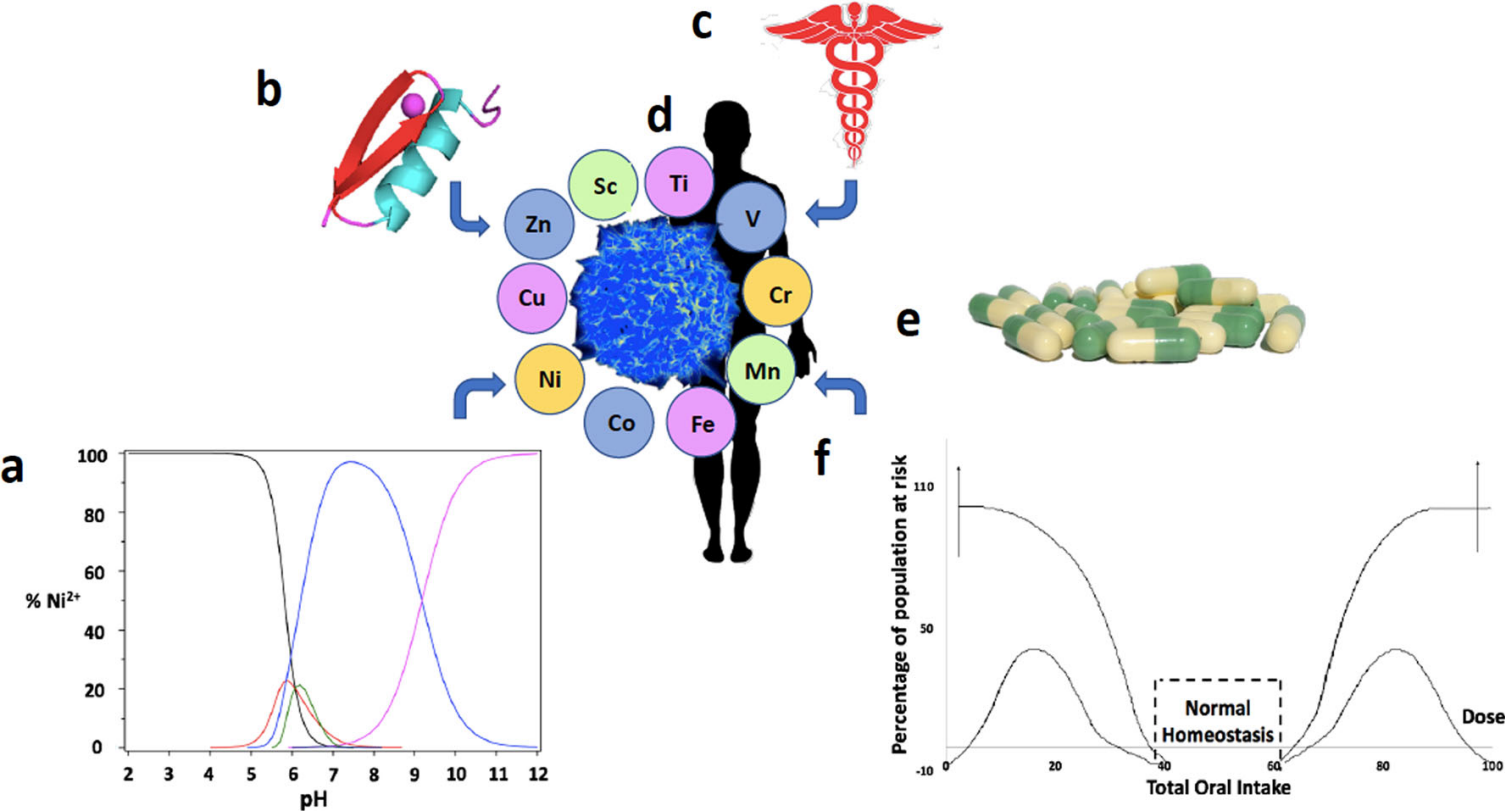
Schematic representing the chemistry and metabolism of the first-row transition metal ions. The speciation diagram reports on the forms of Ni^2+^ at a changing pH^5^ (**a**), Zn^2+^ ion bound to the zinc finger motif (**b**), the ten first-row transition metal ions in cells and human beings (**c**) are responsible for the health of the human (**d**), but may need to be supplemented (**e**) in order to achieve oral homeostasis (**f**). **a** Adapted with permission from ref. ^5^ **b** and **e** were prepared by the authors. **c**, **d** and **f** were reproduced with modifications: Caduceus, Eliot Lash from Wikimedia Commons; Healthy human T-cell, NIAID from http://www.nih.gov; Essential Metal Uptake diagram, Van Cleave et al. from MDPI (ref. ^2^)/under the Creative Commons Attribution-Share Alike 3.0 Unported license (https://creativecommons.org/licenses/by-sa/3.0/deed.en).

The chemistry of all first-row transition metals is very sensitive to their environment^6^. In the presence of water, each metal ion forms hydrated ions which undergo pH and concentration-dependent chemistry that is dictated by the presence of metabolites, proteins, and other biological components (Fig. 1a). It is important to recognize that redox active metal ions do not exist as free ions in cells^7^. As a result, these metals undergo speciation chemistry governed by the metal ion’s oxidation state, the local pH, the ionic strength, and the stability of metal complexes with biological molecules^6,8^. Depending on the specific conditions, several of the metal ions form multinuclear species in aqueous solution, and as such many activities and functions will not be linear but very sensitive to concentrations and association with biomolecules. Appreciation of classical speciation chemistry increases when it becomes obvious that the identification of components in the system demystifies poorly understood processes in biology^9^.

Typically, the first-row transition metal ions are bound to proteins in the cellular system^10^. More than 30% of the proteins in the genome bind metal ions, and some of these bind the metal ions with such a high affinity that the metal ion is difficult to separate from the protein. Protein complexation to a metal ion prevents the redox formation of undesired reactive oxygen species (ROS) by the metal ion. This, however, requires that the bound metal ions are utilized by transfer of the metal ion to a target protein. Processes involving metal transfer reactions can be difficult to study because the reacting metal peptide complex is very likely to have very similar spectroscopic signatures to the product metal peptide complex^11^. Importantly, changes in affinities of the metal ions in different oxidation states are important in facilitating cell uptake and transfer reactions. Manganese, iron, cobalt, and copper are the four essential elements that exhibit rich redox chemistry under physiological conditions. These metal ions are associated with multiple enzymes, and are involved in many cellular redox processes^9,12–14^.

Iron is essential for human life as hemoglobin and as an intricate part of respiration enzymes, particularly those containing hemes^12–14^. The chemistry of both Fe(II) and Fe(III) is important for binding and function of many other redox proteins, including non-heme proteins. Ligand coordination to iron is important to allow the redox cycling without the involvement of Fenton chemistry and generation of uncontrolled ROS at the cellular level^11^. Similarly, copper is involved in function of many redox enzymes as well. Cu(I), Cu(II), and Cu(III) are bound to many proteins and cellular components with high affinity^7,9^. Although manganese and cobalt have less prominent roles than iron and copper, they too are involved in specific processes essential for life. For example, manganese is a cofactor for the enzyme superoxide dismutase responsible for scavenging ROS. Cobalt is bound to a heme in vitamin B_12_ which is the only vitamin to contain a metal ion. Studies of these systems continue to be important. An increased consideration of the metal coordination chemistry will benefit the insights into these systems and uncover new details about their modes of action. Studies with zinc, which is the fifth essential element and the only non-redox active cation, are also important because zinc has either a structural or functional role in more than 300 different proteins^13,14^. One role of zinc is in maintaining the folding of the DNA-binding domains of eukaryotic transcription factors including zinc finger transcription factors. Recent investigations have demonstrated numerous previously unknown activities including hormone-like activities which highlights the notion that even non-redox active ions can have crucial roles in biological systems. The large number of clinical trials involving zinc shown in Table 1 demonstrates the interest in this metal. Importantly, future investigations of these metals in biological systems should be done in the context of metal coordination chemistry within and outside the range of normal homeostasis.

**Table 1:** Clinical trials involving compounds containing first-row transition metals registered with the National Institutes of Health (NIH).

Transition metal	Total clinical trials	Active^a^ clinical trials	Conditions/disease	Interventions/procedure
Scandium	4	2	Melanoma/gingival recession	Isolated hepatic perfusion (IHP)/Laser procedures
Titanium	540	134	A wide range of Ti-based implants	Surgical insertion
Vanadium	12	2	Preeclampsia/prediabetes/cancer	Oral administration
Chromium	256	62	Type 2 diabetes, coronary artery disease, obesity, HIV, polycystic ovary	Oral administration
Manganese	73	17	Vitamin and micronutrient deficiency	Oral administration
Iron	3234	776	anemia, iron deficiency, obesity, type 2 diabetes	Oral administration
Cobalt	299	67	B-cell lymphoma, coronary artery disease, osteoarthritis, melanoma, coronary artery stenosis	Oral administration
Nickel	118	28	Malocclusion, knee osteoarthritis, malignant glioma, impacted tooth, sickle cell disease	Orthodontic arch wire, EXD-959 Bracket System, Surgery, Oral administration
Copper	393	83	Menkes disease, contraception, cancer, amyotrophic lateral sclerosis, HIV, vitamin and mineral deficiency, Wilson disease, vitamin D deficiency	Oral administration, surgery
Zinc	1797	182	HIV, sepsis, hemophilia B, dental pulp necrosis, Mucopolysaccharidosis II, vitamin A and D deficiency, fluoride poisoning, zinc deficiency, cancer, type II diabetes, type 1 diabetes	Oral administration

Taken from https://www.clinicaltrials.gov

^a^“Active” includes funded trials that are either recruiting or completing data analysis.

Three additional elements have some reported beneficial effects include chromium, vanadium, and nickel. Chromium, considered to be an essential metal for some time, is probably the most controversial element in the periodic table^1,8^. Careful speciation studies have shown that Cr(III) is not as inert as previously believed and can convert to Cr(V) and Cr(VI) ions which are highly toxic^8^. Like chromium, both beneficial and toxic effects have been reported for vanadium and nickel. For many years, vanadium compounds were developed as insulin enhancing agents. Current studies focus on using vanadium-based compounds as anticancer agents which include flavonoid vanadium complexes^2^ and coordination complexes for immunotherapy applications^15^. Nickel appears to be an important part of the microflora in the human gut where it is a cofactor for the enzyme urease^2^. On the other hand, Ni-containing alloys often used in jewelry are known to cause an allergenic response in about 30% of women. This application is countered by to the use of Ni-containing alloy implants added to mend broken bones and represents an example of beneficial and toxic effects of this metal^2^.

Titanium is a first-row transition metal ion that has no known biological function despite being readily transported in the human blood where it readily binds to proteins such as human serum albumin. Titanium metal is a common component of alloys ranging from dental implants to orthopedic prosthetics and many clinical trials have been completed and others are underway. However, even for this supposedly non-toxic element, there are some reports that the metal alloys are not completely stable; some cationic forms of titanium leach from the metal surfaces^13^. The biological and potentially toxic response of each system should be carefully considered, particularly since such a large number of clinical trials are ongoing with this element (Table 1). Scandium is non-essential to human health and has no known biological function in the biosphere. Its low abundance has precluded many studies until recently. Potential applications of scandium are currently being investigated in two clinical trials (Table 1).

Essential nutrients for human health include the 13 known vitamins A, C, D, E, K, and the eight B vitamins (thiamine (B1), riboflavin (B2), niacin (B3), pantothenic acid (B5), pyroxidine (B6), biotin (B7), folate (B9), and cobalamin (B12). Unlike minerals, vitamins in biological systems can be metabolized to carbon dioxide and water while metal ions must be removed by excretion. The bioprocessing of these metal ions and their recycling remain a complex matter, which require an evaluation of their speciation chemistry. Unfortunately, studies integrating metal speciation with pharmacokinetic and pharmacodynamic properties of metal ions are also quite costly^13,14^. Nonetheless, metabolism provides additional avenues for formation of active biological substances. That is, if the ligand bound to a metal is changed during bioprocessing, a new complex with a different coordination and speciation chemistry is formed which may also have beneficial biological activity and significantly prolong the effects of the originally administered therapeutic^9^.

Finally, we wish to point out the utility of these elements as therapeutic and diagnostic agents. To document their use, we have tabulated the number of completed and active clinical trials that have been carried out with these ten elements (Table 1). Since five of the first-row transition metals are essential elements, many of their applications are related to bringing the concentration of these elements into the normal concentration range so it is neither too low or too high, thus causing disease. Accordingly, a large number of clinical trials are associated with iron, zinc, copper, cobalt, and manganese. Many applications of titanium and nickel relate to their physical properties and concern their use as alloys in implants. Development of therapeutic and diagnostic agents continues because of the increasing need to monitor and cure diseases^16^. However, technical advances change the requirements for the agents, as illustrated by the recent report where the compound’s reactivity was considered an advantage because injections were made directly into the tumor, and the reactivity of the compound affects cancerous tissue^17^. Multiple uses of the first-row transition metal ions in medicine are already in place in the clinic and other promising uses are being developed with the potential of improving human health.
